# Genome-wide analysis of allele-specific expression of genes in the model diatom *Phaeodactylum tricornutum*

**DOI:** 10.1038/s41598-021-82529-1

**Published:** 2021-02-03

**Authors:** Antoine Hoguin, Achal Rastogi, Chris Bowler, Leila Tirichine

**Affiliations:** 1 Institut de Biologie de l’Ecole Normale Supérieure (IBENS), Ecole Normale Supérieure, CNRS, INSERM, PSL Université Paris, 75005 Paris, France; 2 Université de Nantes, CNRS, UFIP, UMR 6286, 44000 Nantes, France; 3 Present Address: Corteva Agriscience, The V Ascendas, Atria Block, 12th Floor, Madhapur, Hyderabad, 500081 India

**Keywords:** Epigenomics, Gene regulation, Genomics, Genetics, Molecular biology

## Abstract

Recent advances in next generation sequencing technologies have allowed the discovery of widespread autosomal allele-specific expression (aASE) in mammals and plants with potential phenotypic effects. Extensive numbers of genes with allele-specific expression have been described in the diatom *Fragilariopsis cylindrus* in association with adaptation to external cues, as well as in *Fistulifera solaris* in the context of natural hybridization. However, the role of aASE and its extent in diatoms remain elusive. In this study, we investigate allele-specific expression in the model diatom *Phaeodactylum tricornutum* by the re-analysis of previously published whole genome RNA sequencing data and polymorphism calling. We found that 22% of *P. tricornutum* genes show moderate bias in allelic expression while 1% show nearly complete monoallelic expression. Biallelic expression associates with genes encoding components of protein metabolism while moderately biased genes associate with functions in catabolism and protein transport. We validated candidate genes by pyrosequencing and found that moderate biases in allelic expression were less stable than monoallelically expressed genes that showed consistent bias upon experimental validations at the population level and in subcloning experiments. Our approach provides the basis for the analysis of aASE in *P. tricornutum* and could be routinely implemented to test for variations in allele expression under different environmental conditions.

## Introduction

Allele-specific expression (ASE) of genes refers to transcriptional imbalance between alleles in non-haploid organisms. ASE therefore deviates from the concept that alleles are expressed in an equal and biallelic manner. Genomic imprinting was one of the very first reported phenomena of ASE^1^. Mammalian imprinted genes are genes that only express from one of the parental genomes. This imbalance is deterministic and parent-of-origin specific. Genomic imprinting has been reported for hundreds of genes in humans and mice. In mammals, X-linked genes also show allele-specific expression^2^. While males are hemizygotes, female cells inactivate one X-chromosome in a process known as X-chromosome inactivation (XCI). In early development, female cells epigenetically inactivate the paternal or maternal X-chromosome in a stochastic manner resulting in a paternal or maternal monoallelic expression of nearly all X-linked genes^2,3^. This process equilibrates X-chromosome gene dosage between hemizygote males and females. Besides X-chromosome linked genes and imprinted genes, widespread allele-specific expression can also be found on autosomal non sex-linked or imprinted genes (‘autosomal’ ASE—aASE)^4,5^.

aASE has been reported extensively in mammalian tissues^6–12^ but also in *Drosophila*^13^, *Arabidopsis thaliana*^14^ and *Candida albicans*^15^. aASE effects are known in certain gene families. It is observed in olfactory receptor genes^16,17^, which is required for proper neuron development. Genes coding for immunoglobulins also show monoallelic expression which provides alternate ways to generate phenotypic diversity for recognition and defense against pathogens^11^. Stochastic allelic switching has been found to be required for antigenic variation in *Trypanosoma brucei* parasites^18,19^. The study of clonal cell lineages has furthermore demonstrated that aASE can be acquired through development and differentiation and can be propagated to the clonal progeny of the cells^7,8,20^. However, recent advances in single cell genomics suggest that aASE effects are more dynamic even within near isogenic clonal cell lines^4,21–23^.

Chromatin signatures of aASE have been described in mammals and have been found as reliable predictors of allele-specific expression of genes^24,25^. This signature associates repressive marks such as histone 3 lysine 27 trimethylation (H3K27me3) with inactivated alleles and active marks such as histone 3 lysine 36 trimethylation (H3K36me3) with active alleles. Assay for Transposase-Accessible Chromatin with high throughput sequencing (ATAC-Seq) experiments in mice neuroprogenitor stem cells revealed that the promoter of monoallelically expressed genes correlate with the presence of random monoallelically accessible (RAMA) elements^26^. However, a causative regulatory role of epigenetic marking on aASE genes has never been proven.

aASE is not restricted to animals and plants and may be a pervasive feature in diatoms. It has been hypothesized to provide an advantage over haploid algal genomes for fine tuning gene expression in response to environmental triggers. aASE was first reported in the polar diatom *Fragilariopsis cylindrus*^27^. In this study, the authors revealed that more than 25% of genes with highly diverged alleles are differentially expressed under environmentally relevant conditions including low and high temperature, prolonged dark and iron depletion. Around 66% of these genes showed allelic imbalance in at least one experiment, strongly suggesting that allelic imbalance results from species adaptation to new environments. In diatoms, homoeologous gene expression bias (HEB) was furthermore described in the allodiploid and highly oleaginous diatom *Fistulifera solaris*^28^. In this naturally occurring hybrid species, HEB is widespread with 61% of homoeologous genes showing differential expression (expressed allele ratios > 2). During the process of oil accumulation, HEB followed a logic of sub-genome preference dependent on the sub-metabolic pathways studied. This is in line with the hypothesis of hybrid adaptation and in this peculiar case oil accumulation.

In a recent study that compared whole genome sequences (WGS) of different ecotypes of the model pennate diatom *Phaeodactylum tricornutum,* we revealed an extensive map of polymorphic sites clustering ecotypes in different haplotypes^29^. *P. tricornutum* is also the most studied model diatom for which large sets of RNA-sequencing data in different controlled culture conditions are publicly available*.* Moreover, this is the only diatom for which a comprehensive epigenetic landscape has been drawn^30,31^. Genetic variability in *P. tricornutum* might underlie extensive allele-specific expression and adaptation to specific ecological niches. In the current study, we made use of publicly available whole genome RNAseq data as well as polymorphism information in the *P. tricornutum* reference strain Pt18.6^29^ to identify, characterize and quantify allele-specific expression patterns. We validated the level of expression imbalance between alleles on 28 genes. We found that strong allelic expression bias was scarce (1% of genes) but stable in *P. tricornutum* while 22% of genes displayed more pervasive and less stringent allele-specific expression patterns in standard culture conditions. These results provide a methodology for interpreting the consequences of aASE in this ecologically important group of organisms. Our work can be extended as well to other species of the Stramenopile group of eukaryotes to which diatoms belong, which includes numerous other lineages of phytoplankton as well as important plant pathogens.

## Results

### Allele-specific expression in *P. tricornutum*: method and in silico characterization

In order to detect allele-specific expression in *P. tricornutum*, we isolated allele-specific read counts from RNAseq and genomic data previously generated^29^ (“Materials and methods”, Supplementary File 2). We searched for genes with differential allele expression bias focusing on data generated for the reference accession of *P. tricornutum*, namely the Pt18.6 line. We quantified allelic imbalance as a function of allele read counts and read depth of a given single nucleotide variant (SNV) within protein coding sequences using the protocols detailed in Rastogi et al.^29^ and Rastogi et al.^32^ (“Materials and methods”, Supplementary File 2). We computed average percent allele frequency bias (AFB) as allele-specific genomic bias and average percent allele expression bias (AEB) for allele-specific bias in mRNA levels (“Materials and methods”, Supplementary File 2).

Low % AFB and AEB values correspond to genes with low bias in allele frequency/expression while higher percentages correspond to genes with allele-specific expression. Duplicated polymorphism would be expected to give more than 30% AFB (> 2:1 allelic ratio) due to overrepresentation of one allele in genomic sequencing. For functional analysis of genome wide bias of allele-specific expression, we thus excluded genes with AFB > 20%. We also only considered genes for which multiple SNVs, when possible, showed concordant allele-specific expression bias. We further grouped all genes into three categories based on AEB thresholds.

1. ‘Biallelic expressed’ genes (BAE) with low percent expression bias [AFB(%) ≤ 20; AEB (%) ≤ 20].
2. ‘Allele-specific expressed’ genes (ASE) with moderate percent expression bias [AFB(%) ≤ 20; 20 < AEB(%) ≤ 60].
3. ‘Monoallelic expressed’ genes (MAE) with high percent expression bias [AFB(%) ≤ 20, and AEB(%) > 60].

**Figure 1:**
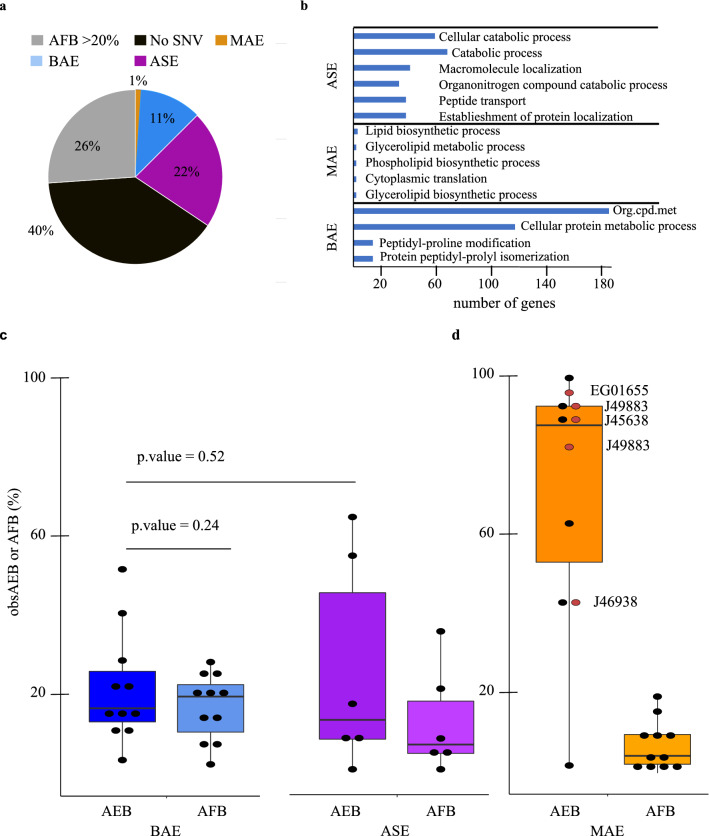
Characterization of allele-specific expression in the model diatom *P. tricornutum*. (**a**) Number of genes in allele-specific expression categories as a percentage of total genes in *P. tricornutum.* (**b**) Examples of the most specific and abundant gene ontologies (biological processes) in ASE, BAE and MAE genes based on TopGO analysis (see also Supplementary Dataset 2). Org.cpd.met = Organic nitrogen compound metabolism. (**c**) Box plot representation of obsAEB (cDNA) and obsAFB (gDNA) values as per pyrosequencing results for a subset of MAE, BAE and ASE SNV. For each SNV, AEB(AFB) was extrapolated from observed difference allelic frequencies. We also named and labelled by red dots the MAE genes tested in subclonal experiments (see Fig. 2).

As a result, 1395 (~ 11%) genes were categorized as ‘BAE’, 2662 (~ 22%) genes were characterized as ‘ASE’, and 129 (~ 1%) genes were denoted ‘MAE’ (Fig. 1a). A total of 4809 (40%) genes did not have any SNVs that could be used to distinguish transcripts according to alleles, while 3207 (26%) genes could not be included in the analysis as their AFB was superior to 20% (Fig. 1a). Accordingly, among 588 genes with copy number variations in the corresponding *P. tricornutum* ecotype ‘Pt1’ (‘file S1’ of Rastogi et al.^29^) only 164 fall within the ASE category, 45 within the BAE category and 2 have monoallelic expression (data not shown). We did not find any compelling associations between gene categories and selective pressure as between 91 and 95% of BAE, ASE and MAE genes are neither under balancing nor constrain selection in the *P. tricornutum* populations described in Rastogi et al.^29^ (data not shown). A summary of all SNVs analysed can be found in Supplementary File 1. AEB (%) values for genes in MAE, ASE, and BAE categories can be found in Supplementary Dataset 1.

### Experimental validation and function of allele-specific expression in *P. tricornutum*

The function of allele-specific expression in diatoms is debated. We thus asked whether BAE, ASE and MAE genes were enriched for any biological process using QuickGo and topGO^33^ tools. We kept biological processes enriched at Fisher p.value < 0.05. We found that BAE genes are specifically enriched for very general cellular protein metabolic processes as well as organic nitrogen biosynthesis processes (Fig. 1b, Supplementary Dataset 2). Interestingly, ASE genes are enriched for genes involved in related catabolism processes including proteasome subunit proteins and autophagy protein families (Supplementary Dataset 2). ASE genes are also enriched in intracellular protein transport, exocytosis and endocytosis processes (Fig. 1b, Supplementary Dataset 2). This is shown for synaptobrevin protein family genes (Supplementary Dataset 2). MAE genes showed a poor level of GO enrichment that must be put in perspective of their underrepresentation in Pt18.6. MAE genes are enriched in a small subset of biological process of lipid metabolism (Fig. 1b, Supplementary Dataset 2). They were also found enriched for genes with hydrolase activity function (Supplementary Dataset 2). The molecular function associated with structural component activity/structural constituent of ribosome and cofactor activity are enriched within BAE genes (Supplementary Dataset 2). Ion binding functions as well as catalytic activities are highly enriched in genes with moderate allele-specific expression (Supplementary Dataset 2).

Our in silico analysis identified a substantial amount (22%) of genes with moderate allele-specific expression which suggests that, although nearly complete bias is scarce (~ 1%), there is nonetheless a frequent less stringent fluctuation of allelic expression in *P. tricornutum*. Allele expression bias could solely be observed due to random effects (as transcriptional bursting^34^). We thus assessed relative allelic expression of individual selected MAE, BAE and ASE genes using a RT-PCR-pyrosequencing approach (“Materials and methods”, Supplementary File 2) and extrapolated observed AEB and AFB values (obsAEB and obsAFB). When several polymorphisms were present in a gene, we monitored (whenever possible) the corresponding SNVs within the same cDNA pyrosequencing read range. We also averaged % allelic differences between multiple SNVs within genes as in the in silico analysis. In total, we tested 6 ASE, 11 BAE and 11 MAE genes in 1 to 3 biological replicates of propagated Pt18.6 cell populations. As internal controls, we also quantified the allelic genomic bias for each SNV to account for technical variations. The list of MAE, ASE and BAE genes tested, and the number of biological replicates performed on both cDNA and gDNA can be found in Supplementary Dataset 3. Examples of pyrosequencing results can be found in Supplementary Fig. 2).

BAE, ASE and MAE genes have on average observed allele genomic bias below 20% (Fig. 1c). At the mRNA level, BAE genes have an average obsAEB of 21.3%, which is higher than the in silico threshold. A total of three BAE genes show moderate expression bias. However, the distribution of obsAEB and obsAFB does not differ (Student test, p.value > 0.05). Four out of six ‘ASE’ category genes display *bona fide* biallelic expression profiles (Fig. 1c). The distribution of obsAEB of ASE genes does not differ from the distribution observed for BAE genes (Student test, p.value > 0.05). In conclusion, we could not confirm that moderate allelic variations were *bona fide* events upon experimental validation. This suggests that genes with medium bias of expression are probably more dynamic and that our in silico experiment is not a good approximation of subtle changes in allelic expression. Monoallelic genes however are concordant upon experimental validation. The obsAEB median for MAE genes is equal to 85% which strongly supports in silico predictions (Fig. 1c). Nonetheless, two MAE genes have moderate bias in expression in vivo and 1 MAE gene is a biallelically expressed gene (Fig. 1c). Our data show that near absolute monoallelic expression occurs on a small subset of genes with fixed allelic imbalances in populations of *P. tricornutum* cells.

**Figure 2:**
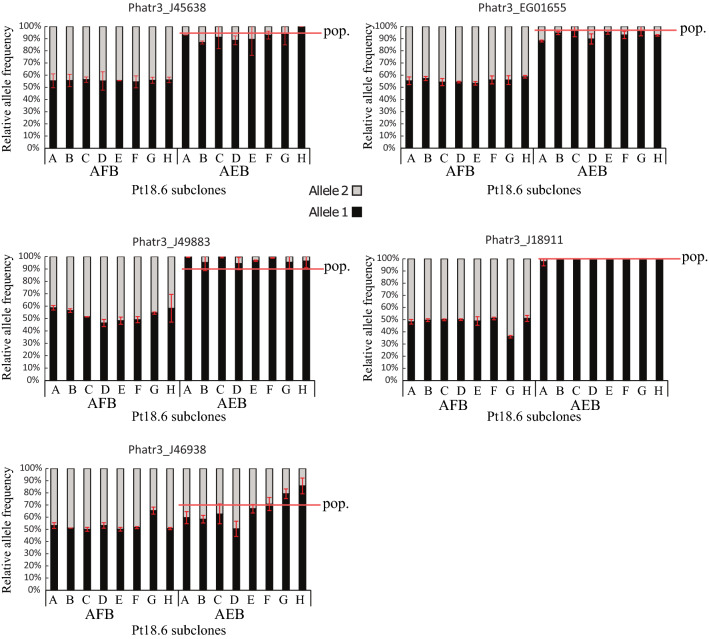
Allele-specific expression is stable upon clonal propagation in *P. tricornutum*. Pyrosequencing results for MAE genes in Pt18.6 subcloned populations (A to H). We represented allele frequency % for each SNV as given by pyrosequencing values. By default, alleles are named ‘allele 1’ and ‘allele 2’, and ‘allele 1’ is the most expressed allele. Error bars represent the standard deviation between 2 technical replicates. SNVs are the same as in table of Supplementary Dataset 1 for the corresponding genes. For each SNV we represent the observed allele frequency (extrapolated from obsAEB) at the population level (Fig. 1) in cDNA by a red line. J45638: 6-pyruvoyl tetrahydropterin synthase; EG01655: unknown function; J49883: glycosyltransferase; J18911: aminoacyl-tRNA synthetase; J46938: NnrU domain containing enzyme.

Variations in allelic expression could reflect clone-specific transcriptional variations. To test this hypothesis, we subcloned Pt18.6 by low density seeding on Enhanced Sea Artificial Water-agar (ESAW-1.5%AGAR) plates. Isolated colonies were further propagated as axenic cultures and pyrosequencing was performed on a subset of MAE genes identified in the previous section. Our results are shown in Fig. 2 as the relative allele frequency for each SNV and each gene in each sub-clonal population. Four genes showed consistent monoallelic bias in clonal populations of *P. tricornutum*. These genes are involved in protein translation regulation and cell metabolism (Fig. 2). In conclusion, MAE genes that show near complete allelic expression bias in silico and in vivo tend to have consistent bias in expression between the population level and the sub-clonal level, strongly suggesting that monoallelic expression is stable through mitotic division in *P. tricornutum*. Phatr3_J46938 has a more contrasted allelic imbalance. In the original experiment (previous section) J46938 has a lower bias in expression than expected in in silico experiments (obsAEB = 40%). It is biallelically expressed in clones A, B and D and is monoallelically expressed in clone H (clone G showed no bias compared to gDNA controls for this experiment). Other clones show moderate biases in expression. It is possible that genes with moderate in vivo AEB have higher subclonal variation than genes with *bona fide* monoallelic expression.

### Epigenetic marks and allele-specific expression

**Figure 3:**
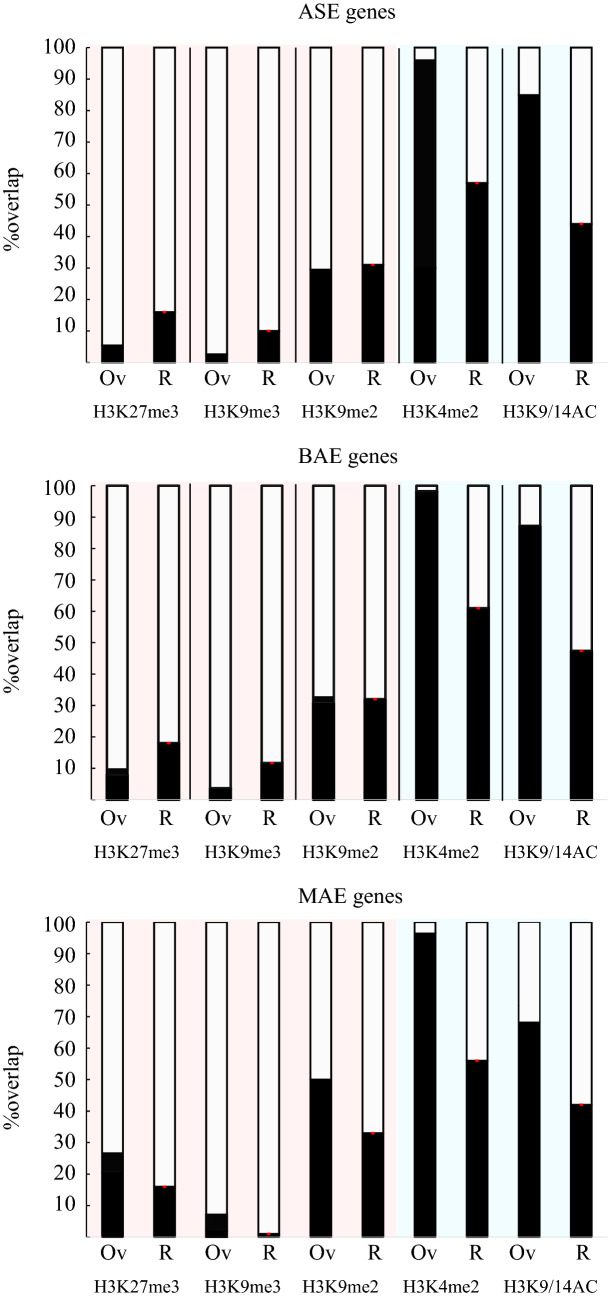
Association of epigenetic marks and allele-specific expression in *P. tricornutum*. Percentage overlap (‘Ov’) between annotated genes within the MAE, ASE, and BAE categories and H3K27me3, H3K9me3, H3K9me2, H3K4me2 and H3K9-14AC peaks. ‘R’ represents mean %overlap over 100 iterations between each gene categories and a random histone peak positioning. Overlap were calculated using R software^43^ R version 3.6.1 (2019-07-05) (https://cran.r-project.org/bin/windows/base/old/3.6.1/) and the genomation R package computation^44^.

We asked whether we could associate allele-specific expression with specific epigenetic marks. Using available chromatin immunoprecipitation data^30^ we computed the overlap between BAE, ASE and MAE genes with repressive ChIP-seq peaks of H3K27me3, H3K9me2 and H3K9me3 as well as H3K4me2 and H3K9/14AC associated with active transcription (Fig. 3). As a comparison, we computed genomic overlap between genes and histone modifications but randomizing the position of histone peaks within their respective chromosomes. We found that all categories of genes share the same epigenetic landscape. Compared to their respective randomized association, MAE, ASE and BAE categories of genes show low overlap with the repressive histone marks H3K27me3 and H3K9me3 that usually associate with transposable elements in *P. tricornutum*^30^. We found nonetheless a modest enrichment of H3K9me2 peaks at MAE genes compared to ASE and BAE genes. MAE genes also show less comparative overlap with H3K9/14AC marks. The enrichment for H3K9me2 marking may highlight the repressive state of one of the allele at MAE genes.

## Discussion

The very first descriptions of autosomal allele-specific expression were from studies of clonal populations of human and mouse cells using whole genome sequencing technologies, including micro-array and next generation sequencing strategies. Between 2 and 14% of autosomal genes were found with monoallelic gene expression not related to genomic imprinting in mouse^9^ and human lymphoblastic cells^6^, in mice fibroblast^9^ as well as in human neural stem cells^10^ and in mice neuro-progenitor stem cells^7,8^. In these studies, some genes showing aASE in one clonal population do not necessarily show the same imbalance in other populations of clonal cells or cell types. While discrepancies between studies can be interpreted by the various technical biases and thresholds used to categorize monoallelic gene expression and allele-specific expression in general, the independent findings of identical bias genes in mESC^7,8^ were key to demonstrate that allelic effects are *bona fide* clonally stable events in mammals. Our study shows that only 129 (~ 1%) of all protein coding genes in *P. tricornutum* can be considered as *bona fide* allele-specific genes, i.e., displaying monoallelic expression and being stable through mitosis. In laboratory conditions, *P. tricornutum* populations are composed of near isogenic colonies of clonal cells that only reproduce by asexual mitotic division in a controlled and standardized environment. Monoallelic gene expression in *P. tricornutum* hence cannot be interpreted as imprinting. Therefore, the observed MAE in *P. tricornutum* matches the definition of aASE as described in mammalian cells.

In *P. tricornutum*, for MAE genes we found that the gene ontology enrichment signal was too weak to draw a general conclusive role for allelic imbalances in *P. tricornutum*. The function, if any, of monoallelic expression must therefore be determined on a case by case basis. BAE genes are involved in very general and homeostatic functions of cellular biology such as ribosome constitution and organic nitrogen metabolism. In contrast ASE genes are involved in catabolism*.* As we showed that allelic imbalances were not fixed in ASE genes, the questions of why dynamic aASE occurs at these genes and whether this is linked to innate clonal variability and phenotypic variations or not should be further investigated. Our data suggest that complete monoallelic expression is mitotically stable in subcloning experiments when maintained under the same and constant environment and is thus likely not due to random transcriptional noise. In *F. cyliindrus*^27^, extensive aASE was observed when comparing transcriptomes generated in very contrasting growth conditions such as prolonged darkness, hinting for a role of aASE in the environmental stress response of *P. tricornutum.* It is thus possible that aASE is an integral part of the gene regulatory network of diatoms. In that regard, a recent computational study described gene co-regulatory networks making advantages of 187 RNAseq datasets generated in *P. tricornutum* cultures in varying growth conditions^35^. The in silico investigation of allele-specific effects within gene networks of *P. tricornurum* cultures subjected to variable growth conditions followed by experimental validation should retain much attention and is the scope of future studies in the field. Our subcloning experiments are only a snapshot of the heterogeneity/homogeneity of the original cell population. The recent advances in single cell whole genome sequencing allowed a new step forward in the understanding of the underlying process of aASE^21^. To our knowledge, no single cell experiments have been performed in any diatom cells. A genome wide picture of clonal monoallelic expression and genetic divergence must be formally established in our subcloning experiments. The possibility that aASE could be involved in adaptation still faces the question of the clonal heterogeneity of this cellular response (being epigenetic or genetic by nature). This was not ruled out by the studies reported in either *F. cylindrus*^27^ or *F. solaris*^28^. We thus strongly suggest that any experiments evaluating the impact of aASE upon changing environments in diatoms should be interpreted with care in several sub-populations of clonally isolated cells for genes with AEB > 60%. *F. solaris* show higher level of aASE compared to *P. tricornutum* that correlates with strong allele-specific nucleotide diversity due to hybridization events^28^. As for *F. solaris*, it is discussed that aASE at divergent haplotypes might be linked to the probable hybrid nature of *F. cylindrus*^36^. The study of polymorphisms between *P. tricornutum* ecotypes suggested that admixing also occurred within natural populations of *P. tricornutum*^29^. It would be interesting to study aASE variations between *P. tricornutum* ecotypes in the light of the diversity of the spots from where they were collected, thus environmental conditions likely acting as drivers for differential expression of alleles.

It is possible that we underestimate or overestimate the extent of monoallelic expression in *P. tricornutum*. As reported in yeast hybrids^37^, compensatory effects at the posttranslational level could buffer transcriptional imbalances. We did not explore this aspect. Mass spectrometry data are starting to emerge in *P. tricornutum*^38,39^. Future studies should consider exploring the extent of protein variants in the bulk *P. tricornutum* translatome.

The mechanisms of onset and maintenance of aASE are elusive. Studies reported chromatin signatures at aASE genes notably in mice^24,26^. In *P. tricornutum*, the link between allele-specific expression and epigenetic marks does not appear to be straightforward. Other epigenetic factors not investigated in this study and known to play a role in the regulation of allele-specific expression (eg., non-coding RNA) might contribute to promote different expression of alleles. A global pattern of allele-specific epigenetic marks at genes in *P. tricornutum* must be drawn. It is also possible that non-epigenetic factors such as allele-specific transcription factor binding sites and promoter variations as shown in *Fragilariopsis cylindrus*^27^ play a major role in determining aASE in diatoms. In the present study we could not link allele-specific transcriptional variations and non-coding SNVs within promoter or terminator regions. Available genomic sequencing data in *P. tricornutum* were generated using short-length RNAseq reads, which allow deep sequencing coverage for AFB quantifications but provide little to no allelic phase information because they were not assembled de novo. Future studies focused on the combination of Illumina based sequencing reads and the use of long range sequencing technologies such as PacBio sequencing, the Oxford Nanopore Technologies and more recently the Sequel II system^40^, would profoundly deepen our understanding of allele-specific regulation in diatom genomes by providing phased variants in haplotypes with high allelic coverage and corrected mutation rates.

## Conclusion

The wealth of extensive RNA-seq data generated for different purposes in *P .tricornutum* as well as important levels of heterozygous polymorphic sites gave us the opportunity to explore allele-specific effects in the reference diatom species. We confirmed monoallelic expression on a subset of genes. Moderate allele expression bias however could not be assessed with certainty in vivo. Upon clonal isolation, monoallelism was maintained at least for a subset of genes, confirming the clonal propagation of monoallelic expression in diatoms. We also found that moderate expression bias is more dynamic suggesting clonal variability even within near isogenic clonal cells. Our study is the first of its kind to explore monoallelic gene expression in *P. tricornutum* and provides a methodological basis for its in-depth study in the future.

## Materials and methods (see also Supplementary File 2)

### In silico prediction of ASE and AEB

Samples accessions used for in silico analysis of allele-specific expression (NCBI accession IDs): Pt18.6 genome—SRR12160955; Pt18.6 transcriptome—SRX2578671.

Variants in genomic DNA (gDNA) and coding fraction (cDNA) were called using the Genome Analysis Toolkit GATK^41^, with parameters used as previously described^32^. In addition, we further filtered the variants that fulfil the following criteria:

1. The variant is within a protein coding sequence (i.e. removing non-coding variants)
2. The approximate read depth (RD) of a given variant is more than or equal to 20 and 5 in gDNA and cDNA samples, respectively. We fixed these thresholds based on the first peak attained in the read depth frequency distribution (Supplementary Fig. 1) of all the variants in each respective sample.

We estimated average percent allele frequency (AFB—for gDNA bias) and average allele expression bias (AEB—for cDNA bias) per gene and for ‘n’ number of SNVs per gene using the following formula:

$$AFB \; (\%) \; or \; AEB \; (\%)=\frac{\sum_{i=1}^{n}\left(\left|\frac{AD\left(Ref\right)}{RD}-\frac{AD\left(Alt\right)}{RD}\right|\times 100\right)}{N}$$

where N = Total number of heterozygous variants mapped on a given gene; AD (REF) = Allelic read depths of the reference allele; AD (ALT) = Allelic read depths of the alternate allele; RD = Approximate read depth of a given variant.

### *Phaeodactylum tricornutum* culture and accession used for experimental validation of allele-specific expression

For experimental validation, the monoclonal *P. tricornutum* CCMP2561 (Pt18.6) line was obtained from Provasoli-Guillard National Center for Culture of Marine Phytoplankton. Cultures were maintained as axenic in autoclaved and filtered (0.22 μM) Enhanced Sea Artificial Water (ESAW—https://biocyclopedia.com/index/algae/algal_culturing/esaw_medium_composition.php) medium supplemented with f/2 nutrients and vitamins without silica and under constant shaking (100 rpm). Cultures were maintained in flasks at exponential state in a controlled growth chamber at 19 °C under cool white, fluorescent lights at 100 μE m^−2^ s^−1^ with a 12/12 h dark/light photoperiod.

### RNA and gDNA extraction

Pt18.6 cell cultures were grown in flasks to exponential state. Culture growth was followed using a hematocytometer (Thermo Fisher Scientific, Pittsburgh, PA, USA) (Supplementary Fig. 3, Supplementary dataset 4). For RNA and gDNA extraction and pyrosequencing, pellets were collected by centrifugation (10 min–4000 rpm) washed twice with marine PBS (http://cshprotocols.cshlp.org/content/2006/1/pdb.rec8303) for 10 min–4000 rpm, followed by a flash freeze in liquid nitrogen. Total RNA extraction was then performed by classical TRIZOL/Chloroform isolations and precipitation by isopropanol. RNA was DNAse treated using DNAse I (ThermoFisher) as per manufacturer’s instructions. For pyrosequencing, 1ug total RNA was reverse transcribed using the SuperScript III First-Strand (Invitrogen) protocol. DNA extraction was performed using the Invitrogen Easy-DNA gDNA Purification Kit following ‘Protocol #3’ instructions provided by the manufacturer. Extracted nucleic acids were measured using QUBIT fluorometer. RNA and gDNA Integrity were controlled by electrophoresis on 1% agarose gels.

### Pyrosequencing protocol (see also Supplementary File 2)

The pyrosequencing protocol was derived from previously established protocols^42^ and consists in a two-in-one PCR based method preceding pyrosequencing. Briefly, for each SNP, pyrosequencing primers were designed using the “PSQ assay design SW v1.0.6” software (https://psq-assay-design.software.informer.com/1.0/). We subsequently modified the corresponding biotinylated primer sequences to include 5′tailed universal sequences: 5′-GTGACGTACTAGCAACG and 5′-TAGCAGGATACGACTATC for the forward or reverse primers accordingly. The sequencing primer was left unmodified and primers were synthetized by the Eurofins company as non-biotinylated oligos. Two-in-one PCR was performed with biotinylated universal primer USF: 5′[Biotin]-GTGACGTACTAGCAACG; and USR: 5′[Biotin]-TAGCAGGATACGACTATC using GoTaq Flexi DNA polymerase (Promega) according to manufacturer’s protocol with the following modifications. If reverse/forward strand to be biotinylated use 0.4 µM final forward/reverse primer, 0.08 µM final reverse/forward primer and 0.32 µM final USR/USF respectively for one 50 μl PCR reaction. The cycling conditions were set up as the following : 3 min 95 °C—(15 s at 95 °C; 15 s at 58 °C; 30 s at 72 °C) × 7 followed by (15 s at 95 °C; 15 s at 56 °C; 30 s at 72 °C) × 40 – 5 min at 72 °C – 4 °C hold. This will amplify a 100–200 bp biotinylated PCR product in which the target SNV is included. Importantly none of the primer used for pyrosequencing are specific for either one of the allele allowing an unbiased sequencing of both alleles. The PCR product is then sequenced by pyrosequencing on a Pyroseq Q96 device. Pyrograms were analyzed using the provided PyroMark Q96 ID software v1 calling for allele quantification. Oberved AEB and AFB values (obsAEB and obsAFB) were then calculated from relative allelic frequencies as follow $obsAEB \; or \; obsAFB=100\times ABS(\%Allele\; 1-\%Allele \;2)$ with ‘allele 1’ being the more highly expressed allele from pyrosequencing data.

### Gene ontology analysis

Gene ontology enrichments were calculated using the “classic” built-in TopGO^33^ algorithm for QuickGO gene annotations for *P. tricornutum* ASE, MAE and BAE genes compared to all QuickGo annotations of *P. tricornutum* genes. GOs with a corresponding Fisher exact test p.value < 0.05 enrichment were kept for further analysis.

### Overlap between histone peaks and gene categories

Percentage overlaps were calculated using R software^43^ R version 3.6.1 (2019-07-05) (https://cran.r-project.org/bin/windows/base/old/3.6.1/) and the genomation R package computation^44^. Genomic overlap are calculated between gene coordinates and histone peak positions extended 500 bp upstream and downstream to account for promoter and regulatory region marking. To estimate the biological significance of each association, a random overlap with each histone prostranslational modifcation was calculated using the built-in ‘calculateOverlapSignificance’ function for 100 times.

## Supplementary Information

Supplementary Information 1.

Supplementary Information 2.

Supplementary Information 3.

Supplementary Information 4.

Supplementary Figure S1.

Supplementary Figure S2.

Supplementary Figure S3.

Supplementary Dataset S1.

Supplementary Dataset S2.

Supplementary Dataset S3.

Supplementary Dataset S4.
