# Supplementary material for: Genome-wide analysis of allele-specific expression of genes in the model diatom Phaeodactylum tricornutum
Source: Sci Rep. 2021 Feb 3;11:2954. doi: 10.1038/s41598-021-82529-1 (PMC7859220; doi:10.1038/s41598-021-82529-1)
Supplement: Supplementary file 1 — Supplementary Information 1. [file 41598_2021_82529_MOESM1_ESM.docx]

**Supplementary File 1:** snpEFF .vcf file of SNVs found in *P.tricornutum* genome

**Supplementary File 2:** Extended material and method. “Bioinformatics analysis used for the discovery of ASE and MAE in *P. tricornutum*” and “Pyrosequencing procedure for allele frequency bias (AFB) and allele expression bias (AEB) in *P.tricornutum”.*

**Supplementary Fig. 1:** Read depth frequency distribution of SNVs.

**Supplementary Fig. 2**: Examples of pyrosequencing results for three genes with monoallelic expression. E: Enzyme, S: substrate, (ATCG) nucleotide dispensation. The position of the SNV is highlighted in yellow. We show the quantification of obsAEB and obsAFB for the gene Phatr3_EG02090. Figure adapted from the Pyrograms after analysis using the PyroMark Q96 ID software v1 (QIAGEN).

**Supplementary Fig. 3:** Growth of *P.tricornutum* Pt18.6 cells in the culture conditions described in this study. The red arrow indicates the collection time of diatom cells for experimental validations of AEB and AFB. Error bars represent the standard deviation of cell counts in 2 to 3 biological replicates.

**Supplementary Dataset 1:** Tables of ASE, BAE and MAE genes and their AFB (GENOME) and AEB (RNA) values.

**Supplementary Dataset 2:** TopGO tables results of molecular function (TopGO-MF) and biological process (TopGO-BP) gene ontology enrichment in BAE, ASE and MAE genes. For each GO, the number of significant genes and the associated Fischer p.value is indicated. Only GOs with p.value <0.05 are shown. We also present a subset of genes with moderate allele-specific expression involved in protein transport (GO:0015031) and cellular catabolism process (GO:0044248).

**Supplementary Dataset 3**: Primer list and detailed pyrosequencing results for SNVs and gene tested in each category. We present details of SNVs extracted from Supplementary Dataset 1. The most expressed allele for each SNV is shown by a capital letter next to the Phatr3_geneID. The chromosome position and nucleotide coordinate of studied SNV is indicated. The effect of the SNV (Synonymous or Non-Synonymous) on the protein sequence is shown. For the corresponding allele 1, we represent the measured relative allele frequency value in biological replicate in cDNA and gDNA as well as the obsAEB and obsAFB values.

**Supplementary Dataset 4:** Growth of *Phaeodactylum tricornutum* in culture conditions used for obsAEB and obsAFB quantifications. Dynamics of cells counts is shown for 3 biological replicates. Growth rate is calculated as the mean growth rate during the exponential phase for each biological replicate using the differential logarithm formula method between points in the exponential phase.
