# Supplementary material for: Genome-wide analysis of allele-specific expression of genes in the model diatom Phaeodactylum tricornutum
Source: Sci Rep. 2021 Feb 3;11:2954. doi: 10.1038/s41598-021-82529-1 (PMC7859220; doi:10.1038/s41598-021-82529-1)
Supplement: Supplementary file 2 — Supplementary Information 2. [file 41598_2021_82529_MOESM2_ESM.pdf]

Supplementary information for :

Genome-wide analysis of allele-specific expression of genes in the model diatom *Phaeodactylum tricornutum*

Antoine Hoguin[1], Achal Rastogi[1#], Chris Bowler[1] and Leila Tirichine[1,2*]

[1]Institut de biologie de l'Ecole normale supérieure (IBENS), Ecole normale supérieure, CNRS, INSERM, PSL Université Paris 75005 Paris, France

[2]Université de Nantes, CNRS, UFIP, UMR 6286, F-44000 Nantes, France

[#]Current affiliation: Corteva Agriscience™, The V Ascendas, Atria Block, 12th Floor, Madhapur, Hyderabad 500081, India

[*]Author for correspondence at Leila.Tirichine@univ-nantes.fr
