# Supplementary material for: Genome-wide analysis of allele-specific expression of genes in the model diatom Phaeodactylum tricornutum
Source: Sci Rep. 2021 Feb 3;11:2954. doi: 10.1038/s41598-021-82529-1 (PMC7859220; doi:10.1038/s41598-021-82529-1)
Supplement: Supplementary file 4 — Supplementary Information 4. [file 41598_2021_82529_MOESM4_ESM.doc]

**Supplementary File 2**

**Genome-wide analysis of allele-specific expression of genes in the model diatom *Phaeodactylum tricornutum***

Antoine Hoguin, Achal Rastogi, Chris Bowler and Leila Tirichine

**Bioinformatics analysis used for the discovery of ASE and MAE in *P. tricornutum*** (scripts will be provided on demand)

**Samples (NCBI accession IDs)**

1. **Pt18.6 genome** (re-sequenced, WGS, gDNA) - SRR12160955
2. **Pt18.6 transcriptome** (total RNA, cDNA) - SRX2578671

**Reference based-assembly of Pt18.6 genome (gDNA) and transcriptome (cDNA)** (Please note that some of the following steps were used in Rastogi et al. 2020, ISME is also described in the supplementary file, ‘*file s4*’, of Rastogi *et al.* 2020, *ISME*^1^)

1. Mapping the reads to the reference genome^2^ (reference genome was downloaded from <http://genome.jgi.doe.gov/Phatr2/Phatr2.download.ftp.html>)
   1. We used Bowtie^3^ version 0.12.7 for alignment
   2. Parameters used: -n 2 -X 400
   3. Output: Sequence Alignment Map (SAM) file and Binary Alignment Map (BAM) file

**Small variant Calling (SNPs)**

1. Preparing the sequence alignment map (SAM) for variant calling
   1. We used SAMTOOLS^4^ version 0.1.18 to sort and index the SAM file.
   2. We used BEDTOOLS^5^ version 2.17.0 to estimate the reference genome coverage by the sequence reads
   3. We used PICARD-tools version 1.107 (http://broadinstitute.github.io/picard/) to further prepare the alignment index file for variant calling using MARKDUPLICATES, ADDORREPLACEREADGROUPS, and BUILDBAMINDEX plugins
2. We used GATK^6^ version 2.8-1 to call the small nucleotide polymorphisms/variants (SNP)
   1. Parameters used:

Genotype mode parameter: --genotyping_mode DISCOVERY

Emission confidence threshold parameter: -stand_emit_conf 20

Confidence threshold parameter: -stand_call_conf 30

- 1. Output: Variant Calling File (VCF)

1. We further filtered the variants that fulfils the following criteria:
   1. The variant should be within a protein coding (i.e removing non-coding variants)
   2. The approximate read depth (RD) of a given variant is more than or equal to 20 and 5 in gDNA and cDNA samples, respectively. We fixed these thresholds based on the first peak attained in the read depth frequency distribution (Supplementary Fig.1) of all the variants in the respective sample.
2. Functional characterization of the variants was performed using SNPEFF^7^ and default parameters. The exact command used is ‘SnpEff Phaeo PT186.vcf’.

**Discovery of ASE and MAE**

1. We used only the filtered single nucleotide variants to explore ASE and MAE in *P. tricornutum.*
2. We used in-house scripts to analyze ASE and BAE, based on the principles described below.
3. We estimated average percent allele frequency bias (AFB - for gDNA), and average allele expression bias (AEB- for cDNA bias), per gene and for ‘n’ number of SNV/gene using the following formula:


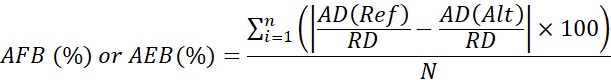


Where, N = Total number of heterozygous variants mapped on a given gene; AD (REF) = Allelic read depths of the reference allele; AD (ALT) = Allelic read depths of the alternate allele; RD = Approximate read depth of a given variant. Reference alleles are defined as alleles mapping on the reference genome as published in Bowler et al^2^.

1. AEB and AFB, thus range from 0 to 100%. Low % AFB and AEB values correspond to genes with low bias in allele frequency/expression while higher percentage correspond to genes with allele specific expression. For functional analysis we excluded genes with AFB>20%, that is to remove duplicated polymorphisms, SNV sequencing bias and genes with multiple discordant SNVs bias. We further grouped all genes into 3 categories based on AEB threshold.
2. ‘Biallelic expressed’ genes **(BAE)** with low percent expression bias [AFB(%)≤20; AEB (%)≤20].
3. ‘Allele specific expressed’ genes **(ASE)** with moderate percent expression bias [AFB(%) ≤ 20; 20<AEB(%)≤60]
4. ‘Monoallelic expressed’ genes **(MAE)** with high percent expression bias [AFB(%) ≤ 20, and AEB(%)> 60].

**Identification of genes exhibiting copy number variations (CNV)**

1. For identifying genes that exhibit CNV in Pt18.6, we used the whole genome read mapping results (BAM file).
2. We used BEDTOOLS^5^ version 2.17.0 to first convert binary version of sequence alignment map file (BAM) to browser extensible data (BED) format.
3. We then used the BED file to estimate the horizontal coverage (how much region of the reference is covered by the reads) and vertical coverage (depth, how many reads are mapped on the reference) of sequence reads on each *P. tricornutum* protein coding gene (Phatr3). Phatr3 gene coordinates can be downloaded from <http://protists.ensembl.org/ Phaeodactylum_tricornutum/Info/Index>.
4. Briefly, we considered Z-score to measure if a gene has more reads mapped compared to an average (mean) number of reads mapped on all the genes.
5. To perform the latter, we first estimated a normalized read depth of each gene to eliminate the following:
   1. The effect of variable size of the sequence libraries
      1. We estimated the proportion (in %) of reads mapped on each gene to the total number of reads in the sequence library.
   2. Variable coverage breadth (horizontal coverage) of a gene
      1. We then multiplied the value from the latter step with the fraction of gene covered by the reads.
   3. Irregularity in sequencing of the genomic regions.
      1. We finally calculated the z-score of each gene using the values already normalized in the previous steps, using the following formula:


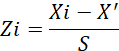


Where, Zi = Z-score of a gene,

Xi = Normalized read count a gene,

X’ = Mean of normalized read count of all the genes

S = Sample standard deviation

Finally, only those genes were deemed as possibly having more copy numbers in the respective genome, where the horizontal coverage was >=0.95 for gene (meaning 95% of the gene is covered by reads), and log2 z-score of the gene is >=2 folds to the average (mean) z-score of read depth of all the genes in a sample.

**Pyrosequencing procedure for allele frequency bias (AFB) and allele expression bias (AEB) in *P. tricornutum***

**Material**

UltraPure DEPC-treated water (ThermoFisher catalogue number: 750024)

SuperScript III First Strand (Invitrogen catalogue number: 18080051)

Invitrogen Easy-DNA gDNA Purification Kit (catalogue number: K180001)

Reagents for RNA (Invitrogen catalogue number: Q10210) and DNA quantification (Invitrogen catalogue number: Q32850)

DNAseI (ThermoFisher catalogue number: 18047019)

TRIzol reagents (Invitrogen catalogue number: 15596026)

Streptavidin Sepharose High Performance beads (VWR catalogue number: 17-5113-01)

GoTaq Flexi DNA Polymerase reagent (Promega catalogue number: M8291)

PyroMark Gold Q96 Reagents (5 x 96) (QIAGEN catalogue number: 972804)

PyroMark Binding Buffer (200 ml) (QIAGEN catalogue number: 979006)

PyroMark Annealing Buffer (250 ml) (QIAGEN catalogue number: 979009)

PyroMark Wash Buffer (200ml) (QIAGEN catalogue number: 979008)

USR: 5’-TAGCAGGATACGACTATC and USF:5’-GTGACGTACTAGCAACG) biotinylated primers

**Equipment**

QUBIT Nano-fluorometer (Invitrogen catalogue number: Q33238)

ThermoCycler (96 well plate)

Eppendorf twin.tec PCR plate 96, skirted (Eppendorf catalogue number: 30128680)

PyroMark Q96 ID System (QIAGEN catalogue number: 9001525) and Software v1.0

PyroMark Q96 Vacuum Workstation (QIAGEN catalogue number: 9001528)

PSQ assay design SW 1.0.6 (https://psq-assay-design.software.informer.com/1.0/)(QIAGEN).

PyroMark Q96 Plate (100) (QIAGEN catalogue number: 979001)

PyroMark Q96 Cartridge (QIAGEN catalogue number: 979004)

**Procedure**

1) Primer design

Material: PSQ Assay Design software (v1.0.6) (QIAGEN)

- Import in the “sequence editor” the genomic (for AFB) or coding/exonic (for AEB) sequence of your gene of interest.
- Edit your sequence to add the alternative base at the SNV(s) present in the sequence using “/” (example: “NNNNNNC/TNNNNN” with “C” the reference allele and “T” the alternate allele). Sequences can also be imported with SNVs following the IUPAC nucleotide code.

A list of primer sets will be generated and organised by quality score for pyrosequencing and PCR amplification for each SNV in the input sequence. Each set is composed of 3 primers: 2 primers for PCR amplification (‘forward’ and ‘reverse’; one should be tagged with biotin) and one “sequencing” primer that will be used during the pyrosequencing process.

- Select the set of primers with the highest quality score for each SNV of interest.
- Export primer sequences as well as the ‘sequence to analyse’ that will be used to determine nucleotide dispensation order. Keep note of the primer that should be biotinylated (we will name them forward -fwd* or reverse -rev*).

We will use universal biotinylated primers to incorporate biotin into the PCR product.

- Edit the 5’end of the forward or reverse biotinylated primer to add a 5’-GTGACGTACTAGCAACG tail for the forward primer or a 5’-TAGCAGGATACGACTATC tail for the reverse primer.
- Order all primers as unmodified oligos

2) Sample preparation

For AEB quantification, RNA was extracted from exponentially grown Pt18.6 cells (~10^6 cells/ml and at least 50ml cell cultures) using TRIzol/chloroform extraction and isopropanol precipitation. Cell pellets were collected by centrifugation (4,000 rpm – 10mins) washed twice with marine PBS (4,000 rpm – 10mins) (http://cshprotocols.cshlp.org/content/2006/1/pdb.rec8303) and flash freeze in liquid nitrogen.

Cell pellets were resuspended in 1ml TRIzol (Invitrogen) and incubated 5min at room temperature. Samples were centrifuged 5min at 12000g and the supernatant was collected and mixed by vortexing with 250µL of chloroform. After 5min incubation at room temperature in chloroform, samples were centrifugated at 12000g for 15min and the aqueous phase was carefully retrieved and put into a clean RNAse free Eppendorf tube. Total RNA is precipitated with 1ml isopropanol for 10 min at room temperature or at -20°C overnight. Prior to cDNA synthesis, RNA was DNAse treated using DNAse I (ThermoFisher) as per manufacturer’s instructions. 1ug of total RNA was used for reverse transcription using SuperScript III First-Strand (Invitrogen) as per manufacturer recommendations. Total volume of cDNA was brought to 100µL with 80µl of DEPC water.

For AFB quantification, 1-2µg genomic DNA was extracted from exponentially grown Pt18.6 cells (~10^6 cells/ml – use between 10ml to no more than 50ml of cultures) using Invitrogen Easy-DNA gDNA Purification Kit following ‘Protocol #3’ as per manufacturer’s recommendation. RNA and gDNA quality are assessed on 1% agarose gels and quantified using a QUBIT fluorometer.

3) PCR and biotin incorporation

Two in one PCR reaction is performed using GoTaq Flexi DNA polymerase (Promega)

**PCR mix:**

5µL of cDNA generated in 2) for AEB; 10-100ng of gDNA for AFB

10µL - GoTaq buffer 5X

1µL - dNTP 10mM

0,4µL - nonbiotinylated tailed-primer F* (or R*) (10µM)

1,6µL - 5’[biotin]USF (or R) primer (10µM)

2µL - non-tailed reverse (or forward) primer (10µM)

0,25µL GoTaq (5u/µL)

DEPC H20 to 50µL

PCR reaction was performed using Eppendorf twin.tec PCR plates.

**PCR program**

95°C 3 minutes

95°C 15 sec

58°C 15 sec 7 cycles

72°C 30 sec

95°C 15 sec

56°C 15 sec 40 cycles

72°C 30 sec

72°C 5 minutes

These steps allow the incorporation of biotin on the forward or reverse strand (via 5’[Biotin]USF or 5’[Biotin]USR)

10 µL PCR products are run on 2% agarose gels and should show a unique 100-200bp band

4) Pyrosequencing procedures

- Create a new SNV run and enter the required instrument parameters such as the PyroMark Q96 Cartridge (QIAGEN) code number.
- Set up your run by creating new SNP index where you can input the “sequence to analyse” for your SNV of interest. Note the estimated usage volume of Enzyme (E-mix), Substrate (S-mix) and nucleotide (A,G,T,C).
- Allow the PyroMark Binding Buffer (200 ml) (QIAGEN) and PyroMark Annealing Buffer (250 ml) (QIAGEN) to equilibrate at room temperature.
- Mix 5 µL Streptavidin Sepharose High Performance beads with 35 µL binding buffer
- Distribute 40 µL of beads-binding solution in each 40 µL PCR reaction well
- Seal the PCR plate and apply constant shaking (1,000rpm) for 10min
- Prepare sequencing mix. Use 1 µL sequencing primer (10µM) in 39 µL PyroMark Annealing Buffer (250 ml) (QIAGEN) for each PCR well to be sequenced.
- Distribute 40 µL of sequencing mix per well in a PyroMark Q96 Plate.
- Place the Eppendorf twin.tec PCR plate (your biotin-labelled PCR product mixed with streptavidin beads) and the PyroMark Q96 Plate (containing the sequencing primer mix) on the vacuum workstation according to manufacturer’s instruction.
- Fill (~ 90ml) the gutters of the vacuum workstation with 70% ethanol, denaturation solution (0.2 µM NaOH), 1X PyroMark Wash Buffer (QIAGEN) and distilled water according to manufacturer instructions.
- Proceed to beads capture, denaturation and washing as per manufacturer instructions
- Incubate the PyroMark Q96 Plate at 80°C for 2min and cool down at room temperature
- Fill the cartridge with the required amount of PyroMark Gold Q96 Reagents and place it into the Pyromark Q96 ID apparatus.
- Interpret sequencing results using the built in PSQ analysis software (v1.0)

Reference

1. Rastogi, A. *et al.* A genomics approach reveals the global genetic polymorphism, structure, and functional diversity of ten accessions of the marine model diatom Phaeodactylum tricornutum. *ISME J.* (2020) doi:10.1038/s41396-019-0528-3.

2. Bowler, C. *et al.* The Phaeodactylum genome reveals the evolutionary history of diatom genomes. *Nature* (2008) doi:10.1038/nature07410.

3. Langmead, B., Trapnell, C., Pop, M. & Salzberg, S. L. Ultrafast and memory-efficient alignment of short DNA sequences to the human genome. *Genome Biol.* (2009) doi:10.1186/gb-2009-10-3-r25.

4. Li, H. *et al.* The Sequence Alignment/Map format and SAMtools. *Bioinformatics* (2009) doi:10.1093/bioinformatics/btp352.

5. Quinlan, A. R. & Hall, I. M. BEDTools: A flexible suite of utilities for comparing genomic features. *Bioinformatics* (2010) doi:10.1093/bioinformatics/btq033.

6. McKenna, A. *et al.* The genome analysis toolkit: A MapReduce framework for analyzing next-generation DNA sequencing data. *Genome Res.* (2010) doi:10.1101/gr.107524.110.

7. Cingolani, P. *et al.* A program for annotating and predicting the effects of single nucleotide polymorphisms, SnpEff: SNPs in the genome of Drosophila melanogaster strain w1118; iso-2; iso-3. *Fly (Austin).* (2012) doi:10.4161/fly.19695.
