# Supplementary figures and images for: Genome-wide analysis of allele-specific expression of genes in the model diatom Phaeodactylum tricornutum
Source: Sci Rep. 2021 Feb 3;11:2954. doi: 10.1038/s41598-021-82529-1 (PMC7859220; doi:10.1038/s41598-021-82529-1)

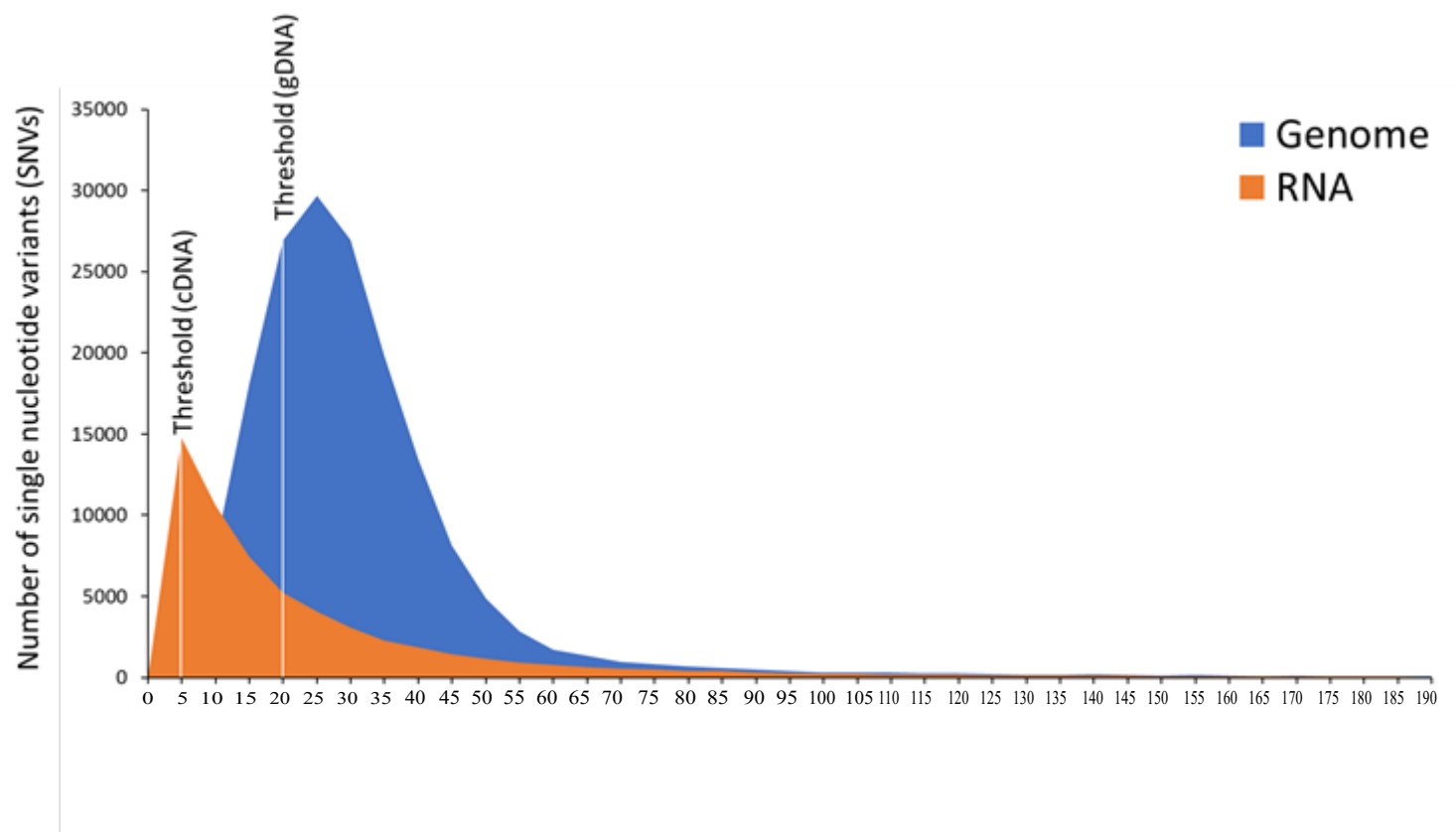

Supplement: Supplementary file 5 — Supplementary Figure S1. [file 41598_2021_82529_MOESM5_ESM.pdf]

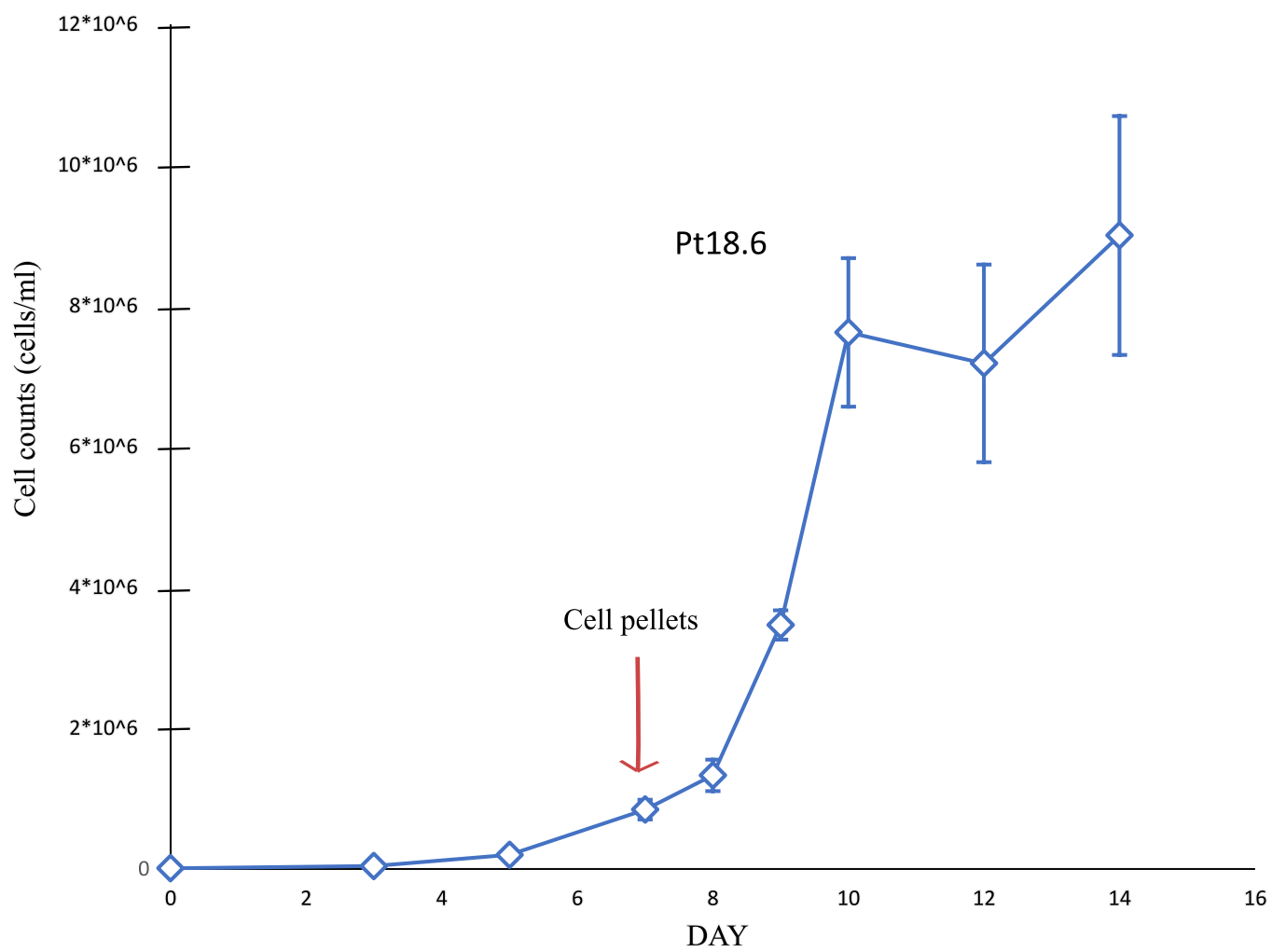

Supplement: Supplementary file 7 — Supplementary Figure S3. [file 41598_2021_82529_MOESM7_ESM.pdf]
