# Supplementary material for: Genome-wide analysis of allele-specific expression of genes in the model diatom Phaeodactylum tricornutum
Source: Sci Rep. 2021 Feb 3;11:2954. doi: 10.1038/s41598-021-82529-1 (PMC7859220; doi:10.1038/s41598-021-82529-1)

Phatr3_EG02090

SNV chr5:377306 Sequence to analyze :GC/TGCCTTT TTCTTTTTCG GGGGTC

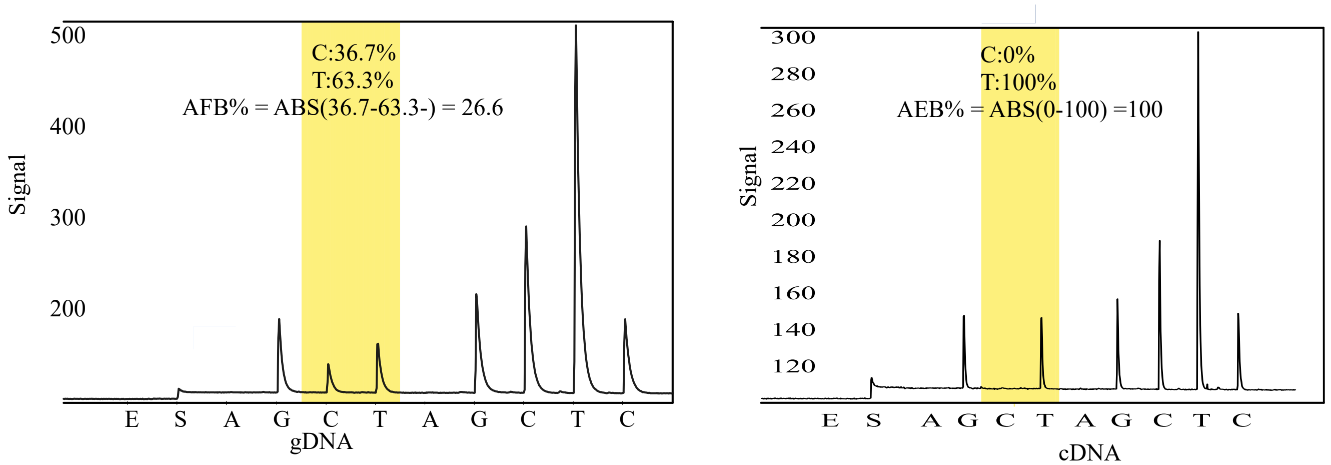

Phatr3_J37136

SNV chr12:240683 Sequence to analyze :CCTCT/ACGC GCGTTCGTTT

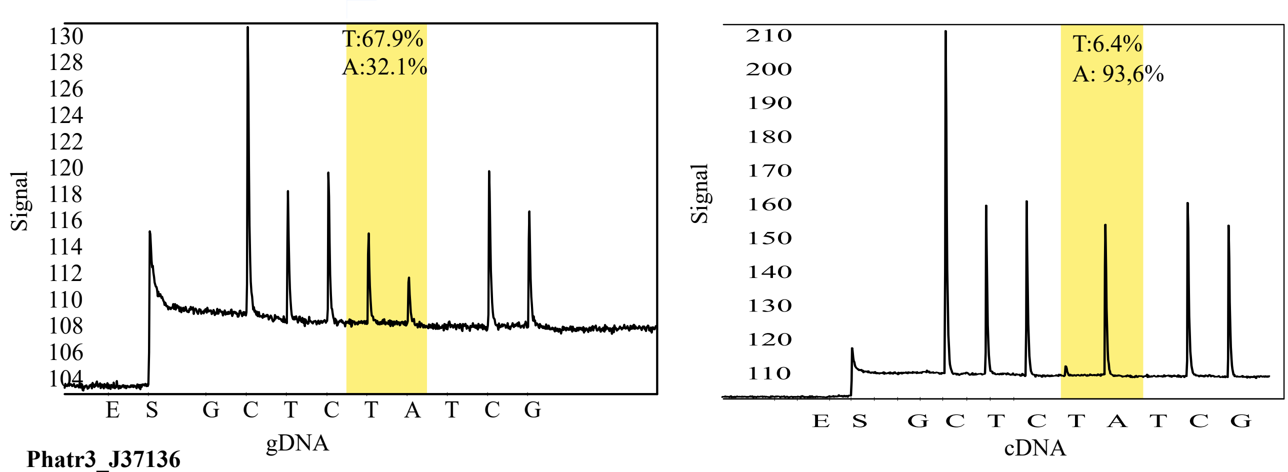

Phatr3_J37136

SNV chr12:241598 Sequence to analyze :GTCA/GTTTG TTCTGCCTTC

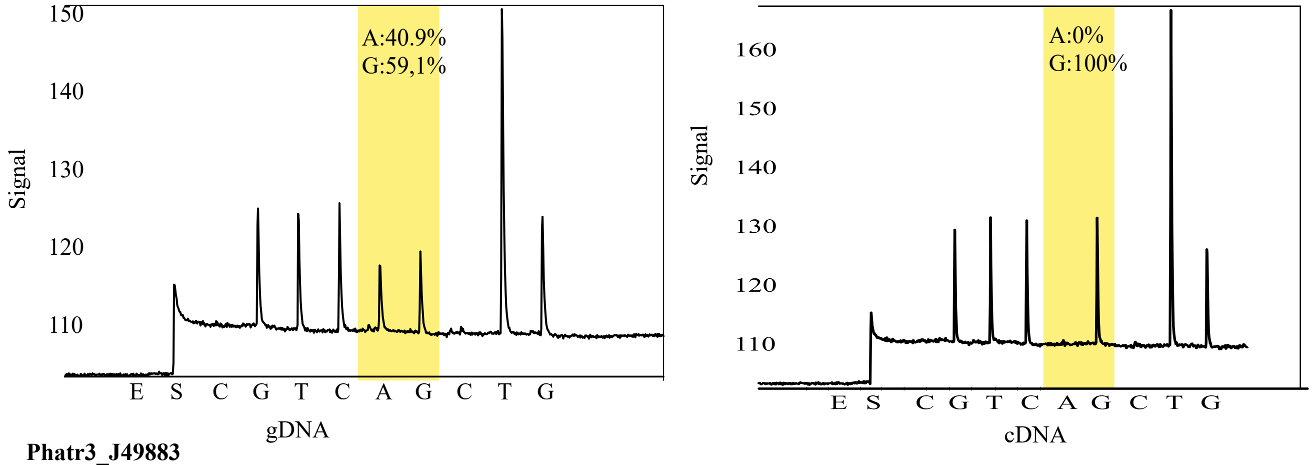

Phatr3_J49883

SNV chr25:185069 Sequence to analyze :CCC/GATACC CAGCATCATC

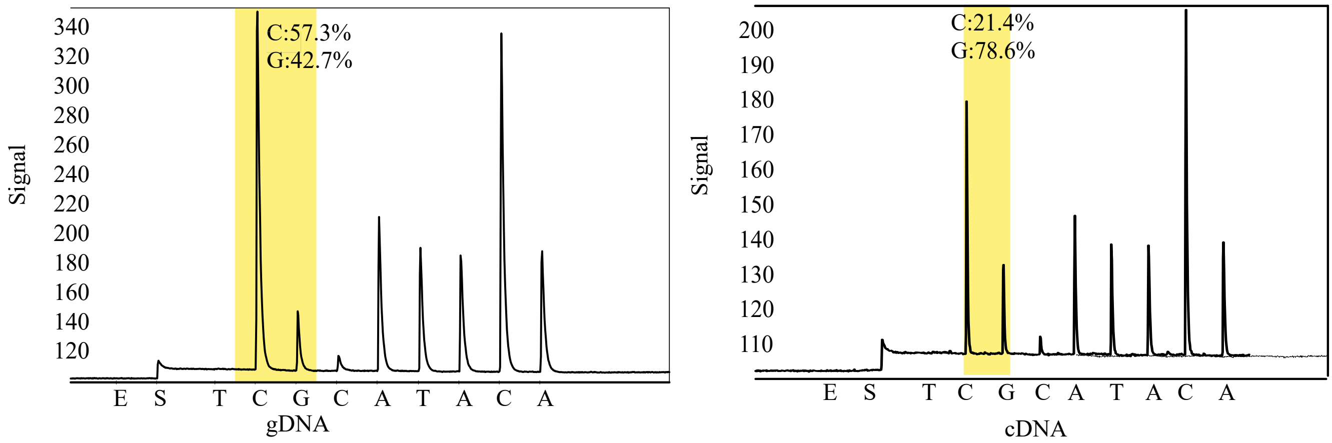

Supplement: Supplementary file 6 — Supplementary Figure S2. [file 41598_2021_82529_MOESM6_ESM.pdf]
